# The one-in-all diagnostic value of ^99m^Tc-MDP bone scan combining with single-photon emission tomography (SPECT)/CT imaging in spinal osteoblastoma

**DOI:** 10.1186/s13018-020-01653-2

**Published:** 2020-05-24

**Authors:** Wenhui Ma, Zhiyong Quan, Jing Wang, Xiangdong Li, Guoquan Li

**Affiliations:** 1 Department of Nuclear Medicine, Xijing Hospital, Fourth Military Medical University, 127# West Changle Road, Xi’an, 710032 Shaanxi Province China; 2 Department of Orthopedic Oncology, Xijing Hospital, Fourth Military Medical University, 127# West Changle Road, Xi’an, 710032 Shaanxi Province China

**Keywords:** Osteoblastoma, Bone scan, SPECT/CT

## Abstract

**Background:**

Osteoblastoma (OB) is an intermediate lesion, which makes the accurate preoperative diagnosis very important. ^99m^Tc-methylene diphosphonate (^99m^Tc-MDP) bone scan and SPECT/CT imaging were evaluated for their diagnostic value in spinal OB.

**Methods:**

This study was a retrospective analysis of patients with spinal OB lesions confirmed by pathology and diagnosed with bone scan and SPECT/CT for preoperative diagnosis from January 2008 to December 2018. The uptake levels of OB on planar bone scan were divided into low, medium, and high groups by visual assessment referring to the uptake of the normal rib, spine, and bladder. X-ray, CT, MRI, bone scan, and SPECT/CT imaging of the patients were analyzed for characteristics summary.

**Results:**

Twenty-five patients were diagnosed for spinal OB (17 males and 8 females with a proportion of 2.1:1), and the average age was 26.8 ± 10.8 years (range 5~59). There were 8 lesions located in the cervical, 6 in the thoracic, and 11 in the lumbar vertebrae. Twenty-four lesions involved posterior elements, especially the pedicles (14/25). Symptoms were predominantly painful with a duration of 18.3 ± 13.9 months (range 0.5~60 months). The lesion size ranged from 9 to 35 mm. All the lesions were low to high uptake in the planar bone scan, and the percentages of low to high levels were 1 (4%), 8 (32%), and 16 (64%) cases.

**Conclusions:**

Spinal OB mainly involved the posterior area, and elderly patients should be considered as well. SPECT/CT combined the characteristics of bone uptake and anatomical features of bone tumors, proving its one-in-all diagnostic value for spinal OB and other osteogenic tumors.

## Introduction

Osteoblastoma (OB) is a rare and primary bone neoplasm which was first described by Jaffe and Lichtenstein in the 1950s, accounting for 1% of all primary bone tumors and around 3% of benign bone tumors [1–3]. OB is commonly located in the spine (35–50%) and usually in the posterior elements. Other frequently involved sites are the femur (16.4%), humerus (7%), and tibia (6%) [2]. OB is histological similar to osteoid osteoma (OO) but differs in size and more aggressive than OO biologically and can infiltrate extraskeletal tissues. When the diameter of lesions exceeds 1.5 cm, the diagnosis is highly suggestive of OB [4–6]. Malignant transformation in 12–25% of lesions has been described in the literature [7, 8]. Spinal OB most commonly affects the posterior aspects of the spine (laminae, pedicles, or spinous processes). The main clinical manifestations are of neurological nature and include progressive focal or radicular pain exacerbated by movement [6, 9]. Surgical resection is the main treatment method with high recurrence rates debated by the subtotal resection [10–12]. The effective treatment of spinal OB relies on precise diagnosis based on a crucial image. However, the radiographic features of OB can differ according to its location and type.

The diagnostic methods for spinal OB mainly include plain X-ray, computed tomography (CT), magnetic resonance imaging (MRI), and bone scan (BS). Plain radiography is usually the first diagnostic approach but has low sensitivity [13]. It often fails to reveal the tumor because of their relatively small size and complex anatomy around the vertebral column. The average delay in diagnosis is approximately 18–24 months in reports [7]. CT can present OB as a sclerotic or osteolytic expansive lesion with a central nidus and surrounding reactive soft tissue change. Sometimes, the presence of bone marrow and soft tissue changes of OB is characterized by a hypointense signal on T1-weighted sequence and high signal on T2-weighted in MRI, whereas ^99m^Tc-methylene diphosphonate (^99m^Tc-MDP) bone scan is commonly used in OB diagnosis because of its osteogenic feature, especially for those with ambiguous or negative radiographic results. It is especially helpful in children since the exact site of pain is difficult to elicit. However, the limitation of the planar bone scan results from false negative. It is hard to differentiate with another osteogenic disease especially in the diagnostic specificity and reduced sensitivity for bone marrow disease. Thus, the specificity of bone scan remains a concern.

By combining functional and anatomical information in a single imaging method, SPECT/CT gained widespread popularity in the last few years and has become a one-stop cancer imaging modality [14]. A few case reports have also demonstrated the usefulness of ^99m^Tc-MDP SPECT/CT for the diagnosis of osteoid osteoma but not OB [15–17]. However, to date, there is no systematic study evaluating the utility of ^99m^Tc-MDP SPECT/CT in spinal osteoblastoma. Therefore, we evaluated the role of SPECT/CT as a one-in-all imaging modality for the diagnosis of spinal OB in this study.

## Materials and methods

Twenty-five patients were confirmed as spinal OB in histopathology and treated from January 2008 to December 2018. All procedures were in accordance with the ethics committee of Xijing Hospital and with the Helsinki Declaration of 1975 (revised in 2008). All patients were investigated by the following imaging resources performed in our hospital: plain X-rays, CT scan, MRI, bone scan, and SPECT/CT. Two experienced radiologists and nuclear medicine physicians reviewed the imaging results.

### Radiotracer injection, planar bone scanning, and SPECT/CT acquisition

The patients were intravenously injected with ~ 10 MBq/kg (260–300 μCi/kg) of ^99m^Tc-MDP for planar BS and SPECT/CT imaging. Images were acquired on SPECT/CT dual-head gamma camera (Symbia T2 or GE670, USA). Parallel-hole and low-energy high-resolution collimators were used with a 140-keV photopeak and a 20% symmetrical window. Delayed planar BS was performed 3~4 h after radiotracer injection. Anterior and posterior whole-body planar images were acquired in a continuous mode with the patient in the supine position (matrix 256 × 1024). The acquisition orbits of SPECT/CT were body contour orbits over 360° arcs, each of 6° for 60 stops. Emission data were acquired for 15 s per stop. The image acquisition matrix was 128 × 128.

### Processing and analysis of images

All studies were uniformly processed with commercially available E.soft software (Siemens, USA) on a Syngo nuclear medicine workstation (Siemens, USA). SPECT images were reconstructed with the Flash-3D software (Siemens Medical Solutions, USA) with 8 subsets and 4 iterations. Subsequently, tomographic slices were generated and displayed as transaxial, coronal, and sagittal slices. SPECT emission images were co-registered and fused with CT images using the object versus the target matrix method.

### Image interpretation

**Fig. 1:**
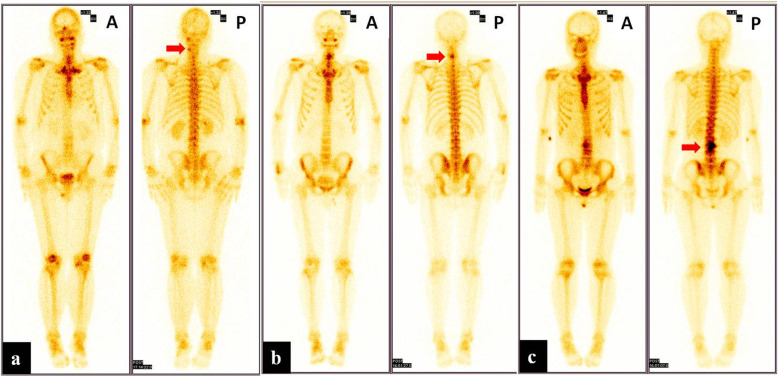
Examples of spinal OB lesion (red arrow) uptake level on a planar bone scan. Low (**a**). Medium (**b**). High (**c**). A and P indicate the anterior and posterior imaging, respectively

X-ray, CT, and MR images were evaluated by two experienced radiologists. The imaging features of the lesions were recorded such as tumor nidus, surrounding sclerosis, signal changes of the lesion, and surrounding tissues. Planar bone scan and SPECT/CT images were analyzed by two experienced nuclear medicine physicians. The readers were aware of the patients’ clinical information but were blind to other imaging findings. The interpreters visually divided the relative uptake of lesions into four levels, negative uptake as the background, low uptake as the ribs, medium uptake as the spine, and high uptake as the bladder (Fig. 1) [18]. For SPECT/ CT, OB was diagnosed based on abnormal focal uptake and SPECT along with specific features on CT.

### Surgical treatment

The procedure was performed under general anesthesia. The surgical method (posterior approach, anterior approach, or combined approach) was selected based on the location and range of the lesion. The posterior approach (PA) was chosen if the lesion involved posterior elements of the spine, such as laminae, pedicles, or the transverse and spinous processes. Anterior approach (AA) and combined approach (AA+PA) should be taken into consideration if the lesion involved vertebral body. The treatment approach, length of stay, duration of the surgery, and blood loss were recorded.

## Results

### Patient characteristics

Twenty-five patients (17 males and 8 females) with a mean age of 26.8 ± 10.8 years (range, 5–59 years) were scanned with the ^99m^Tc-MDP planar bone scan and SPECT/CT to detect spinal OB. Although 80~90% of cases are diagnosed before 30 years of age reported in the literature, 36% of patients (9/25) in our study were diagnosed after 30 years old. There were 8 lesions located in the cervical vertebra, 6 lesions in the thoracic vertebra, and 11 lesions in the lumbar vertebra. According to the location, OB usually involved the posterior area of the spine (24/25), especially in the pedicle of the vertebral arch (14/25). The symptom caused by OB was usually pain with or without numbness or weakness. The duration of the symptom was 18.3 ± 13.9 months (range, 0.5~60 months) depending on the lesion’s location, treatment methods, and individual tolerance level. The characteristics of the patients were demonstrated in Table 1.

**Table 1:**
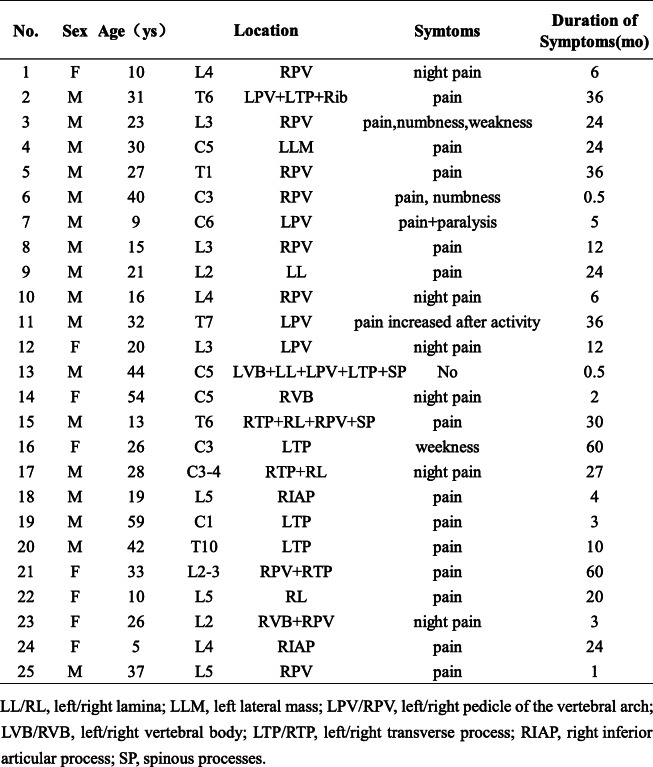
Patient demographics, location of spinal OB, symptoms, and duration of symptoms

### Imaging results

The imaging appearance of OB commonly mimics that of osteoid osteoma. However, it is usually larger in size (> 2 cm) and may have aggressive features according to the World Health Organization definitions published in 2013. The diameter of lesions in this study was from 9 to 35 mm. The imaging features with a detailed description of X-ray, CT, MRI, planar bone scan, and SPECT/CT were shown in Table 2.

**Table 2:**
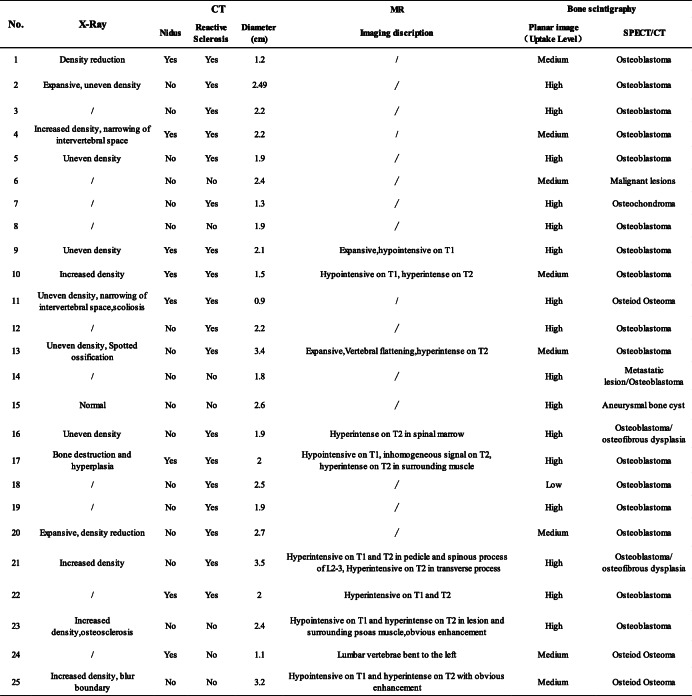
Imaging findings with more specific descriptions of the abnormalities

**Fig. 2:**
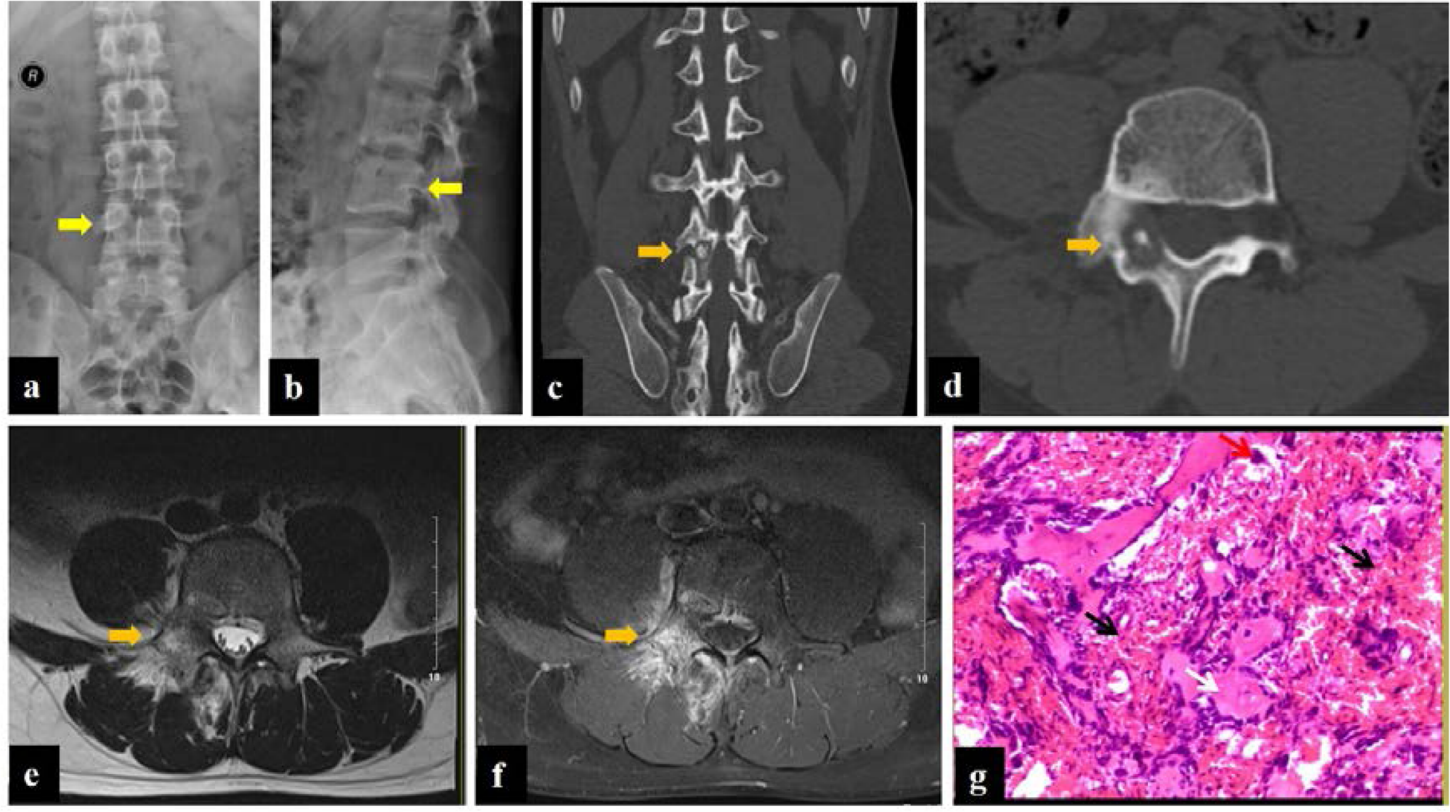
Patient no. 10, male, 16 years old. Anteroposterior (**a**) and lateral (**b**) radiograph demonstrated the increased density in the right attachment of L4 (yellow arrow). CT (**c**, **d**) and MRI (**e**, **f**) of the lumbar spine represented a 15.4-mm lesion on the right vertebral arch of L4 with typical features of OB (orange arrow). Note the surrounding bone marrow and paraspinal soft tissue edema on T2W (**e**) and enhanced  T1W/FS sequence (**f**), a typical finding of OB. The histopathology (**g**) displayed osteoblast (red arrow), bone-like tissue and woven bone (white arrow), and loose connective tissue rich in dilated small blood vessels (black arrow), indicating a diagnosis of OB

The radiographic features in classic OB include a well-circumscribed, osteolytic-osteosclerotic lesion with an expansive, scalloped, or lobulated appearance. The detection of OB in the axial skeleton, complex anatomy, and adjacent articular region was limited on account of the superposition of other structures. Therefore, the imaging findings on X-ray usually included uneven density and expansive changes, making it difficult to distinguish from other lesions. CT is considered as the diagnostic modality of choice for tumor detection, which is based on the demonstration imaging features such as central nidus with surrounding sclerosis (Fig. 2). It could accurately show the extent of the lesion, presence or absence of matrix mineralization, location of the lesion (i.e., cortical or medullary) and surrounding bone changes. Sometimes there is erosion of the cortex, and a common finding is the presence of a sclerotic rim representing a reaction from the bone and periosteum. MR may overestimate the aggressiveness of OB, due to the associated marked inflammation or edema illustrated as “flare response” (Fig. 2). MRI features were non-specific with typically low to isointense T1- and T2-weighted signal, decreased signal due to matrix calcification (if present), and intense enhancement representing the highly vascular nature of the lesion.

OB belongs to osteogenic lesions, and all 25 patients were found to have spinal lesions with positive ^99m^Tc-MDP uptake (low~high level) result in BS (Table 2). The number of patients with low-, medium-, and high-level uptake in planar BS was 1 (4%), 8 (32%), and 16 (64%), respectively. SPECT/CT was a much more specific and valuable modality in the differential diagnosis of pathologic osseous conditions. The morphologic CT appearance of the scintigraphic positive lesion could achieve accurate differentiation between benign lesions and metastases based on the whole-body bone scan.

**Fig. 3:**
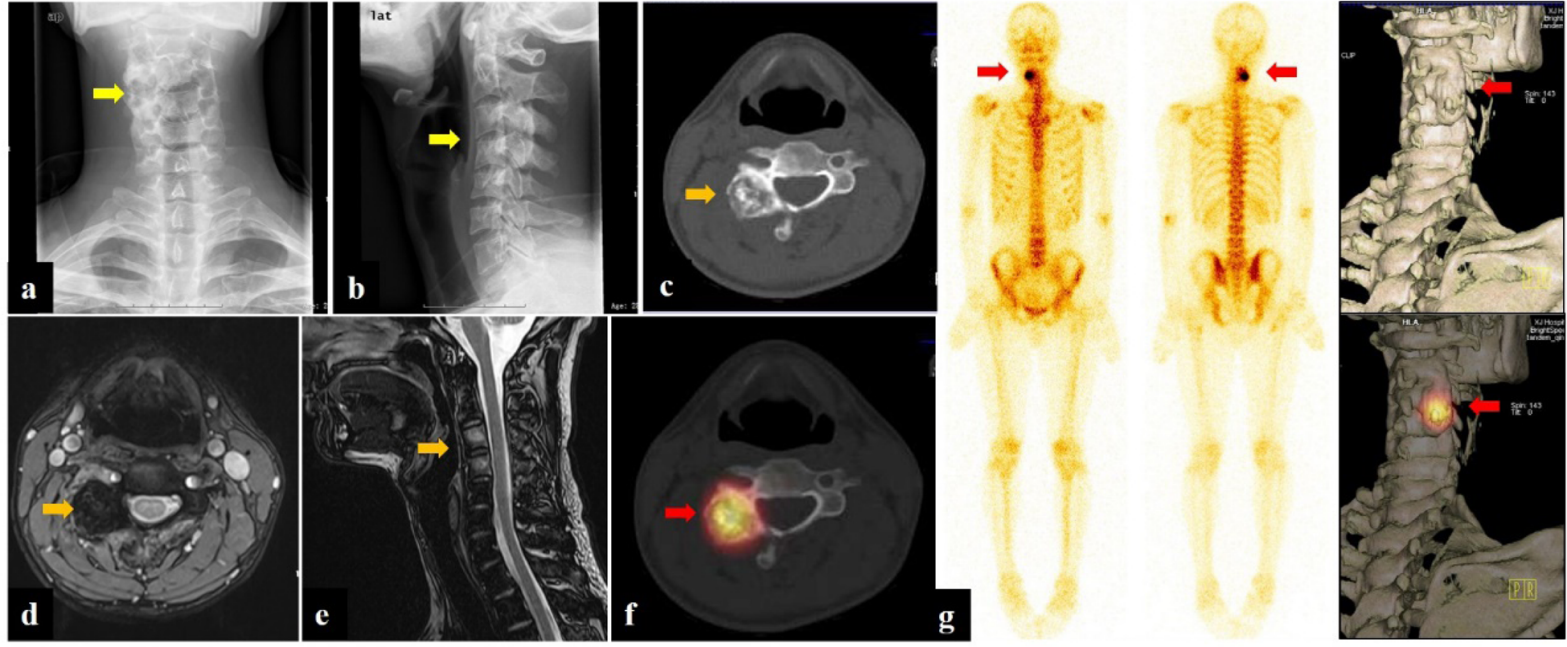
Patient no. 10, male, 16 years old. Planar bone scan (**a**) demonstrated high uptake in the right attachment of the L4 level, indicating strong osteogenesis. SPECT/CT imaging (**b**) and 3D reconstruction images (**c**, **d**) clearly showed the center solid nidus with peripheral osteosclerosis

Malignant characteristics may occur in OB lesions sometimes, such as thinning, erosion of the cortex, and associated soft tissue mass. For example, patient no. 24 was diagnosed with tuberculosis based on CT with atypical imaging features and unclear boundary with surrounding tissue. Meantime, MRI showed defects in the bone lesion and exaggerated peripheral response especially when the bone lesion was small, so the initial diagnosis was left lumbar curvature. Patient no. 17 (Fig. 3) and 21 displayed transverbrate lesions, indicating malignancy, which may also lead to a misdiagnosis as a metastatic lesion.

### Surgical results

The symptoms, operative time, blood loss, and treatment approach of the patients were summarized in Table 3. The mean operative time was 202.0 ± 22.4 min (range, 120~360 min), and the average blood loss was 656.0 ± 428.2 mL (range, 100~4000 mL). The range of blood transfusion was 370~1420 mL. The mean length of hospital stay was 15.9 ± 5.8 days (range, 8~24 days). The symptoms of the pain were obviously relieved in the majority of the patients immediately after surgery. There were no leakage of cerebrospinal fluid, infectious complications, and neurological injury during the procedure. Histology confirmed OB in all patients after the surgery.

**Table 3:** Brief summary of spinal OB’s surgical treatment

	Value
Length of stay (days)	15.9 ± 5.8
Duration of the procedure	202.0 ± 22.4
Blood loss	656.0 ± 428.6
Treatment approach	PA (23), PA + AA (2)
Red blood cell transfusion (IU)	4 ~ 10
Serum transfusion (mL)	370 ~ 1420

## Discussion

Osteoblastoma occurs rarely in people older than 50 years of age, but sporadic cases of adulthood were described in the literature [2, 7, 19, 20]. In our study, four patients’ age was older than 40 years old, and the oldest age was up to 59 years old. Therefore, OB is not a young person’s exclusive illness. OB should be considered in the differential diagnosis of bone lesions of the spine in adulthood and in the elderly (which includes osteoblastic metastases like prostate and breast, vertebral hemangioma, osteochondroma, giant cell tumor, chondrosarcoma, chordoma, osteogenic sarcoma, or Paget disease) to reach a diagnosis rapidly and to avoid a delay in the definitive treatment.

**Fig. 4:**
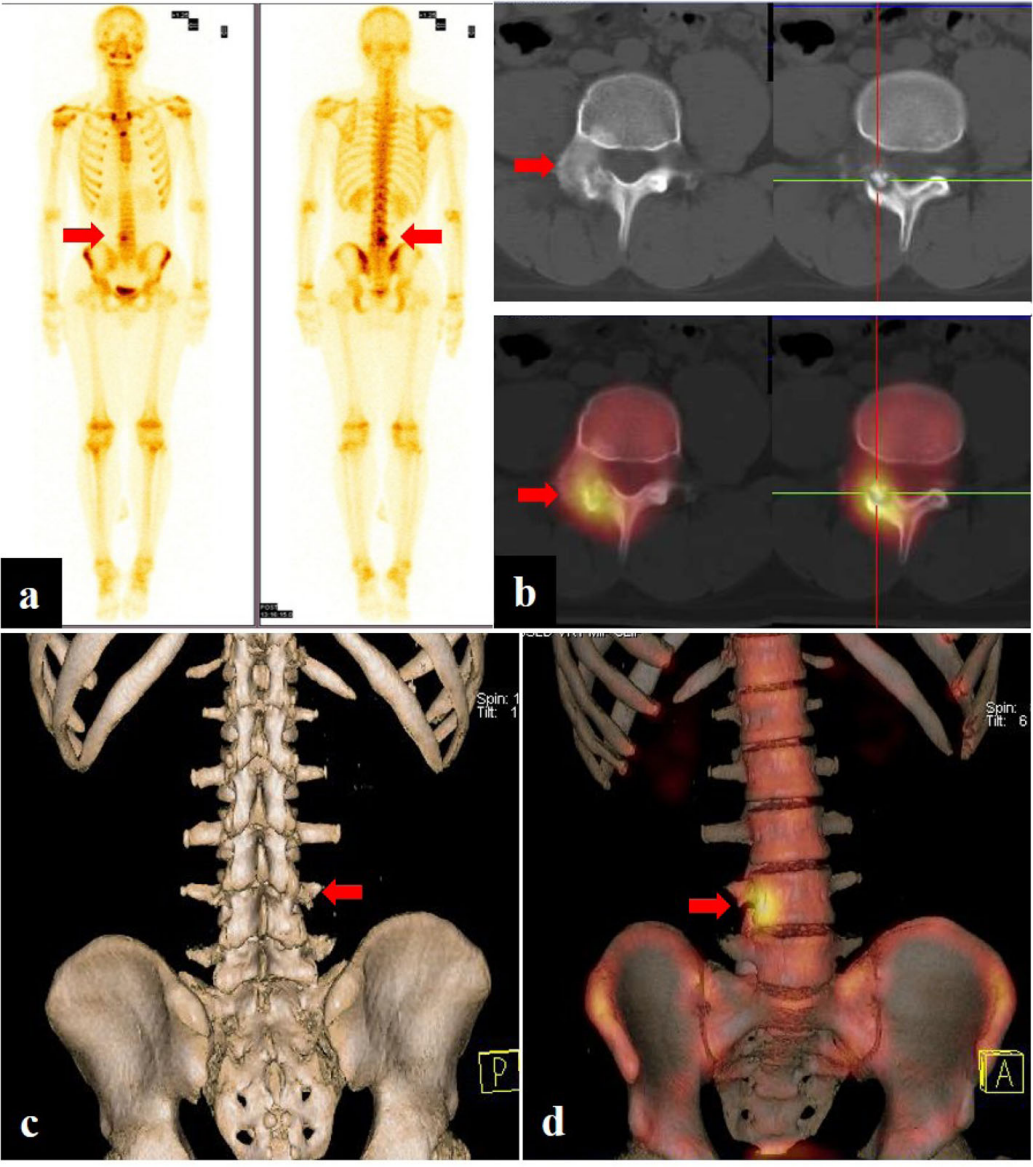
Patient no. 17, male, 28 years old. Anteroposterior (**a**) and lateral (**b**) radiograph demonstrated bone destruction and hyperplasia in the C3–4 level (yellow arrow). CT (**c**) and MRI (enhanced T1W/FS and T2W sequence) (**d**, **e**) of the cervical spine showed a 20.0-mm lesion on the right transverse processes and laminae of C3–4, a typical osteogenic feature of OB (orange arrow). Planar bone scan displayed high uptake around C3–4 cervical vertebral body (**g**), whereas SPECT/CT  (**f**) and 3D reconstruction images showed more details on the lesion and provided more information for orthopedist (red arrow)

CT is best for the observation of the fine structure, the intensity of cortical destruction, and the formation of a bone shell and soft tissue masses. A round or oval lytic lesion with soft tissue or hyperdense nidus surrounded by variable amounts of osteosclerosis was taken as positive imaging features for OB on CT [21]. According to our study, the spinal lesions usually involve the transverse process and the spinous process. OB cases (Fig. 4) may mimic malignancy, particularly when there was expansion, destruction of the bone cortex, new periosteal bone formation, or involving more than one vertebra. The aggressive nature of OB was also well illustrated in a study by Raskas et al., in which 56.6% of OB invaded the epidural space. However, the features occurred in none of 159 OO [22, 23]. If the radiologic findings suggest an aggressive lesion, biopsy should be done, including the periphery of the tumor or the cortical bone surrounding the tumor. MRI has a limited role in primary osseous tumors, because it poorly visualizes the bone marrow which can result in an inaccurate diagnosis of aggressive or malignant lesions [24]. Some patients were misdiagnosed with inflammatory diseases based on the less defined margin between osseous and soft tissues. Prevalent diffusion in young patients is probably due to the greater activity of osteoblasts, but the stromal component could explain the presentation.

Bone scintigraphy combined with CT or MRI becomes the most common method to diagnose and follow up skeletal metastases. However, using hybrid SPECT/CT can improve the specificity in cancer patients by accurately differentiating uptake between benign and malignant sites. Most patients with skeletal muscle metastases generally had markedly widespread involvement which led to an unusual appearance on SPECT/CT imaging. In spite of benign pathologic characteristics of OB, intense glucose metabolism of the tumor on FDG PET/CT suggested a malignancy [25]. Moreover, SPECT/CT provided an accurate identification of tumor viability that was useful for differential diagnosis, treatment planning, and follow-up. Osteoproliferative sclerosis needs to be differentiated from bone infection or osteosarcoma [26]. Both of them can be characterized by extensive bone sclerosis and involving soft tissue change. It was unique to find out whether there is a classical tumor nest. MRI showed a low or mixed signal and needs to be differentiated from chondroblastoma. When OB was associated with an aneurysmal bone cyst, it indicated a hybrid signal in MRI or mixed density on CT. Because of overlapping clinical and pathologic features between OB and OO, differential diagnosis is often unclear and difficult [27]. OB exacerbates pain that is non-responsive to non-steroidal anti-inflammatory drugs, and it usually—but not always—involves axial skeleton. There were certain defects in the lesions according to their diameter or size, such as unclear tumor boundaries, incomplete cortical bones or tumor infiltration through the intervertebral space into the spinal canal, fluid-level or secondary aneurysmal bone cysts, or lesions growing. Whether or not the diameter is bigger than 2 cm, OB should be considered. Aggressive behavior is within the biologic spectrum of OB, and histopathology alone does not appear to be a reliable predictor. Some patients may benefit from SPECT/CT in the following therapy.

The treatment option for OB mainly depends on its size and location. RFA is still a debatable procedure for the management of OB [28]. Surgery represents the first-line treatment in OB because it is the only strategy that ensures complete disease healing. Surgery decreases the risk of local recurrence and enables us to offer the best quality of life in correlation with life expectancy considering the fact that it mainly afflicts young people [29]. Since this study was a retrospective analysis, the incomplete data and low incidence rate of OB made a large number of studies necessary in the future. However, orthopedic oncology surgeons become more and more dependent on the accurate diagnoses of bone tumors with planar scanning and SPECT/CT.

## Conclusions

Spinal OB mainly involved the posterior area, and middle-aged or elderly patients should be considered as well. SPECT/CT combined the characteristics of bone uptake and anatomical features of bone tumors, indicating an excellent way for one-in-all diagnosis and differentiation of spinal OB and other osteogenic tumors. With the popularity of SPECT/CT, it will show great potential for diagnosing the ambiguous bone disease and be helpful for further treatment in the future.

## Abbreviations

^99m^Tc-MDP: ^99m^Tc-methylene diphosphonate
AP: Anterior approach
BS: Bone scan
CT: Computed tomography
MRI: Magnetic resonance imaging
OB: Osteoblastoma
OO: Osteoid osteoma
PA: Posterior approach
RFA: Radiofrequency ablation
SPECT: Single-photon emission tomography
