# Correction to: The one-in-all diagnostic value of 99mTcMDP bone scan combining with singlephoton emission tomography (SPECT)/CT imaging in spinal osteoblastoma

**DOI:** 10.1186/s13018-020-01738-y

**Published:** 2020-06-24

**Authors:** Wenhui Ma, Zhiyong Quan, Jing Wang, Xiangdong Li, Guoquan Li

**Affiliations:** 1 Department of Nuclear Medicine, Xijing Hospital, Fourth Military Medical University, 127# West Changle Road, Xi’an, 710032 Shaanxi Province China; 2 Department of Orthopedic Oncology, Xijing Hospital, Fourth Military Medical University, 127# West Changle Road, Xi’an, 710032 Shaanxi Province China

**Correction to: Journal of Orthopaedic Surgery and Research (2020) 15:181**

**https://doi.org/10.1186/s13018-020-01653-2**

Following publication of the original article [1], it was noted that due to a typesetting error, the captions to Figs. 3 and 4 were mismatched. The caption of Fig. 3 should be put under the Fig. 4 and vice versa.

**Fig. 3:**
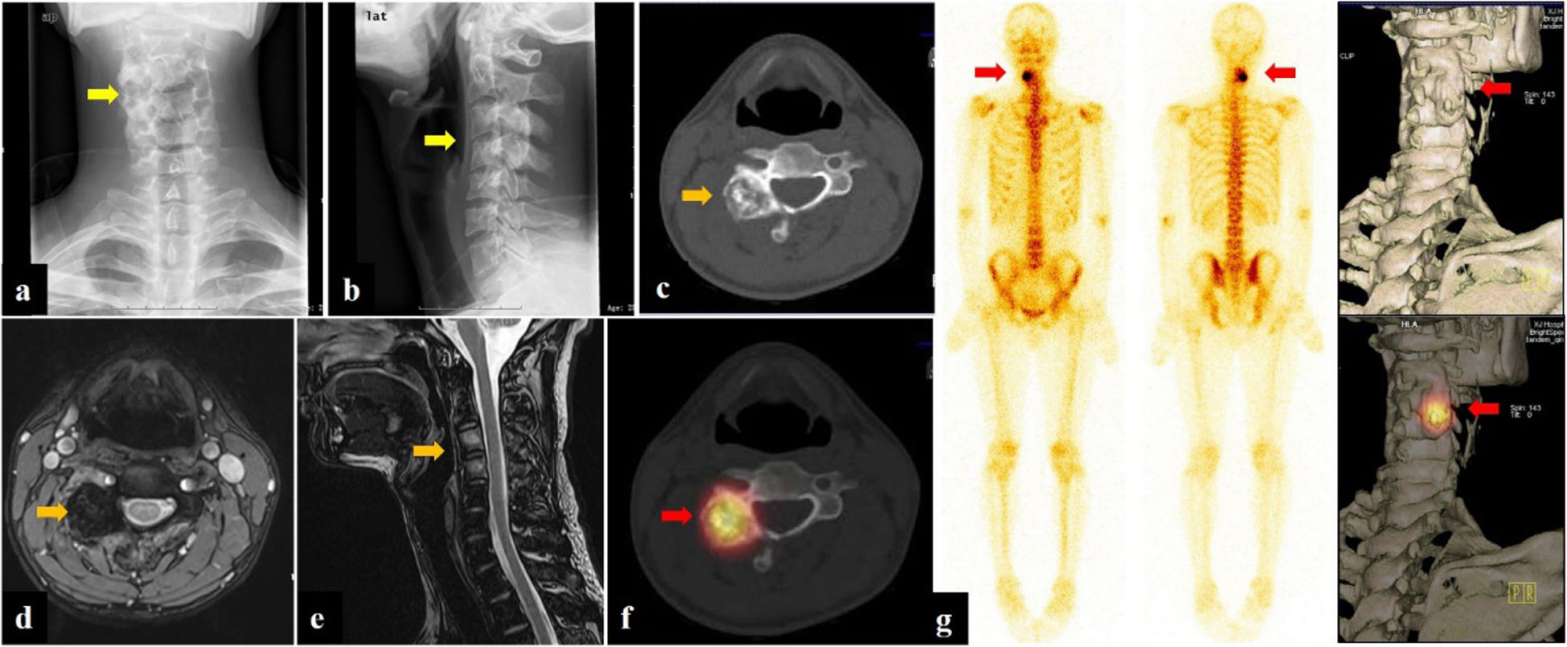
Patient no. 17, male, 28 years old. Anteroposterior (**a**) and lateral (**b**) radiograph demonstrated bone destruction and hyperplasia in the C3–4 level (yellow arrow). CT (**c**) and MRI (enhanced T1W/FS and T2W sequence) (**d**, **e**) of the cervical spine showed a 20.0-mm lesion on the right transverse processes and laminae of C3–4, a typical osteogenic feature of OB (orange arrow). Planar bone scan displayed high uptake around C3–4 cervical vertebral body (**g**), whereas SPECT/CT (**f**) and 3D reconstruction images showed more details on the lesion and provided more information for orthopedist (red arrow)

**Fig. 4:**
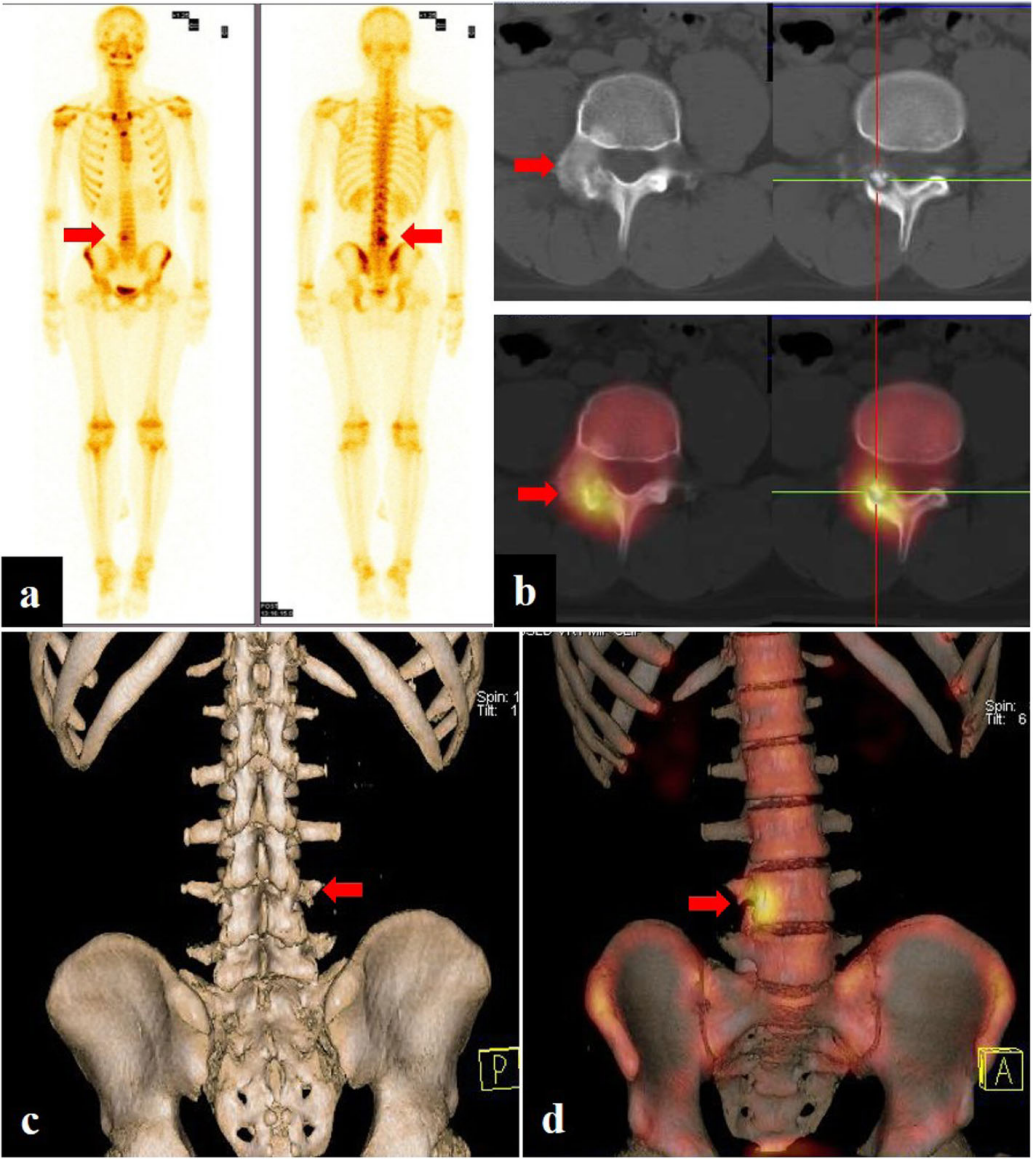
Patient no. 10, male, 16 years old. Planar bone scan (**a**) demonstrated high uptake in the right attachment of the L4 level, indicating strong osteogenesis. SPECT/CT imaging (**b**) and 3D reconstruction images (**c**, **d**) clearly showed the center solid nidus with peripheral osteosclerosis

An error was identified in the Materials and methods section and in Table 3.

The updated Materials and methods section is given below and the changes have been highlighted in bold typeface.

Materials and methods

Twenty-five patients were confirmed as spinal OB in histopathology and treated from January 2008 to December 2018. All procedures were in accordance with the ethics committee of Xijing Hospital and with the Helsinki Declaration of 1975 (revised in 2008). All patients were investigated by the following imaging resources performed in our hospital: plain X-rays, CT scan, MRI, bone scan, and SPECT/CT. Two experienced radiologists and nuclear medicine physicians reviewed the imaging results **respectively**.

The correct Table 3 and the correct figures and captions have been included in this correction.

**Table 3:** Brief summary of spinal OB’s surgical treatment

	Value
Length of stay (days)	15.9 ± 5.8
Duration of the procedure (min)	202.0 ± 22.4
Blood loss (mL)	656.0 ± 428.6
Treatment approach	PA (23), PA + AA (2)
Red blood cell transfusion (IU)	4 ~ 10
Serum transfusion (mL)	370 ~ 1420

The original article has been corrected.
